# Promising effects of exercise on the cardiovascular, metabolic and immune system during COVID-19 period

**DOI:** 10.1038/s41371-020-00416-0

**Published:** 2020-09-17

**Authors:** Isley Jesus, Valentin Vanhee, Therese B. Deramaudt, Marcel Bonay

**Affiliations:** 1 Université Paris-Saclay, UVSQ, INSERM END-ICAP, 78000 Versailles, France; 2 Service de Physiologie-Explorations Fonctionnelles, Hôpital Ambroise Paré, AP-HP, Boulogne, France

**Keywords:** Cardiovascular diseases, Diabetes, Metabolic diseases

## Abstract

With 4 billion people in lockdown in the world, COVID-19 outbreak may result in excessive sedentary time, especially in the population of vulnerable and disabled subjects. In many chronic disorders and diseases including type 2 diabetes mellitus and hypertension, cardiovascular and immune beneficial effects of exercise interventions should be reminded.

Direct metabolic and endocrine link between type 2 diabetes mellitus (T2DM), hypertension, and coronavirus SARS-CoV-2 disease (COVID-19) was recently reported [1]. It is also important to note that with 4 billion people in lockdown in the world, COVID-19 outbreak may result in excessive sedentary time, especially in the population of vulnerable and disabled subjects. Indeed, this population is very dependent on the caregivers in charge of their rehabilitation, since the trip to the patients’ homes may be made more difficult during the outbreak. In many chronic disorders and diseases including T2DM and hypertension, cardiovascular, metabolic and immune, beneficial effects of exercise interventions have been reported [2, 3]. The intensity, volume, and mode of exercise may exert different activation of the hypothalamic-pituitary-adrenal axis, of the autonomous nervous system and of the resulting immunoregulatory hormones that influence immune response. Exercise interventions may affect susceptibility to infection, as they were shown to modify monocytes and lymphocytes distribution, phenotype and cytokine production.

As observed in many other chronic disorders and diseases including atherosclerosis and cardiovascular disease, chronic obstructive pulmonary disease, obesity, and insulin resistance, T2DM is characterized by systemic inflammation and oxidative stress [4]. Animal models have shown that inflammatory cytokines expressed in adipose tissue were involved in obesity-linked insulin resistance. Recently, macrophages were defined as key cells of innate immunity able to regulate metabolic homeostasis and inflammation [5]. They express different anti or pro-inflammatory phenotypes modulating inflammation in metabolic tissues involved in glycemic homeostasis like adipose tissue, liver, pancreas and skeletal muscle. Recently, growing evidences suggest the role of immune system in hypertension and the contribution of immune cells, cytokines, and innate and adaptative immunity in experimental models of hypertension [6]. Angiotensin II, a key hormone of the renin-angiotensin system, is involved in the link between metabolic and immune response in hypertension through the activation of immune cells [7]. Although inflammation plays a central role in the development of T2DM or atherosclerosis and hypertension, anti-inflammatory molecules are not recommended in current therapeutic strategies. Potential candidates targeting tissue-specific inflammation, and minimizing the risks of systemic complications and/or comorbidities, are lacking.

Moderate exercise is known to exert beneficial health effects in patients with chronic disorders and diseases [2, 3]. A Randomized Controlled Trial was conducted to test whether an intensive lifestyle intervention (including 5–6 weekly aerobic training sessions with 30–60 min duration, of which 2 to 3 sessions were combined with resistance training) results in equivalent glycemic control compared with standard care in participants with T2DM [8]. In this 1-year follow-up study, 73.5% of patient in lifestyle group and 26.4% in the standard care group presented a reduction in glucose-lowering drugs. Moreover, 56.3% of the participants in the lifestyle group have discontinued glucose lowering medication, suggesting that exercise may be an efficient treatment. Interleukin-6 produced by skeletal muscle during exercise might improve glucose and lipid metabolism and exert direct anti-inflammatory effects by an inhibition of tumor necrosis factor alpha and by stimulating interleukin-1 receptor antagonist in patients with cardiometabolic diseases [9]. In animal models, exercise was assessed to counteract deleterious cardiovascular effects of anti-inflammatory molecules like dexamethasone (i.e., capillary density decrease in skeletal muscles and arterial blood pressure increase). Jesus et al. [10] showed that 8 weeks aerobic exercise training in rats prevented dexamethasone-induced hypertension and microvascular rarefaction by increasing antioxidant enzymes and improving the balance between apoptotic and angiogenic proteins.

Acute and chronic effects of exercise on immune response have been extensively studied in athletes [2]. When done in excess, heavy exercise is associated with increased risk of illness, attributed to immune dysfunction. Increased inflammatory biomarkers and increased risk of upper respiratory tract infections were observed after acute bouts of intense and prolonged exercise in athletes. During the post-race period, the increased susceptibility was correlated to suppressed salivary immunoglobulin A output, decreased natural killer cell activity and reduced T- and B-cell function. At the opposite, moderate acute and chronic exercise-induced immune changes have been involved in the beneficial effects of physical activity to prevent cancer and cardiovascular diseases development [2, 3, 9]. Immune cells in anti-inflammatory responses are linked to mitochondrial fatty acid oxidation [11]. Recently, improvement of fatty acid oxidation-dependent respiration was reported in peripheral blood mononuclear cells of healthy volunteers with a sedentary lifestyle during low-intensity exercise [12]. Further studies are needed to assess the potential anti-inflammatory effects of low-intensity exercise in patients with cardiometabolic diseases. Concerning global health beneficial effects of low volume leisure-time physical activity, a large observational study showed that 15 min/day for 6 days a week of low-volume activity reduced all-cause mortality by 14%, cancer mortality by 10%, and mortality from cardiovascular disease by 20%, compared to individuals in the inactive group [13].

In animal models of viral respiratory infection, chronic exercise reduced illness severity, viral load and resulted in greater anti-inflammatory effects than acute exercise during influenza infection. Interestingly, epidemiologic data suggest that moderate exercise decreases mortality and incidence rate for influenza infection [14, 15]. Viral respiratory infections, including coronaviruses, induce severe inflammation, which is partially triggered by reactive oxygen species production and dysfunction of the host’s antioxidant defense [16]. Interestingly, exercise interventions may stimulate antioxidant response, particularly via the Nuclear factor erythroid 2-related factor 2 (Nrf2) transcription factor that have been involved in antimicrobial defense and cardiovascular risk in metabolic diseases [17, 18].

In this context, chronic moderate and adapted exercise may be doubly beneficial in T2DM and cardiovascular diseases for preventing inflammation and viral respiratory infection, including coronavirus infection (Fig. 1). High incidence rates of overweight and obese patients are observed in COVID-19 intensive care units and many data suggest that pre-existing comorbidities including hypertension, diabetes, and cardiovascular disease increase severity and mortality rate of COVID-19. Whether exercise training programs, as secondary preventive interventions after healing, would confer immune protection to patients with cardiovascular and metabolic disease compared to sedentary controls, would deserve further investigations. The location of the exercise training programs, indoors or outdoors, should also be assessed.

**Fig. 1:**
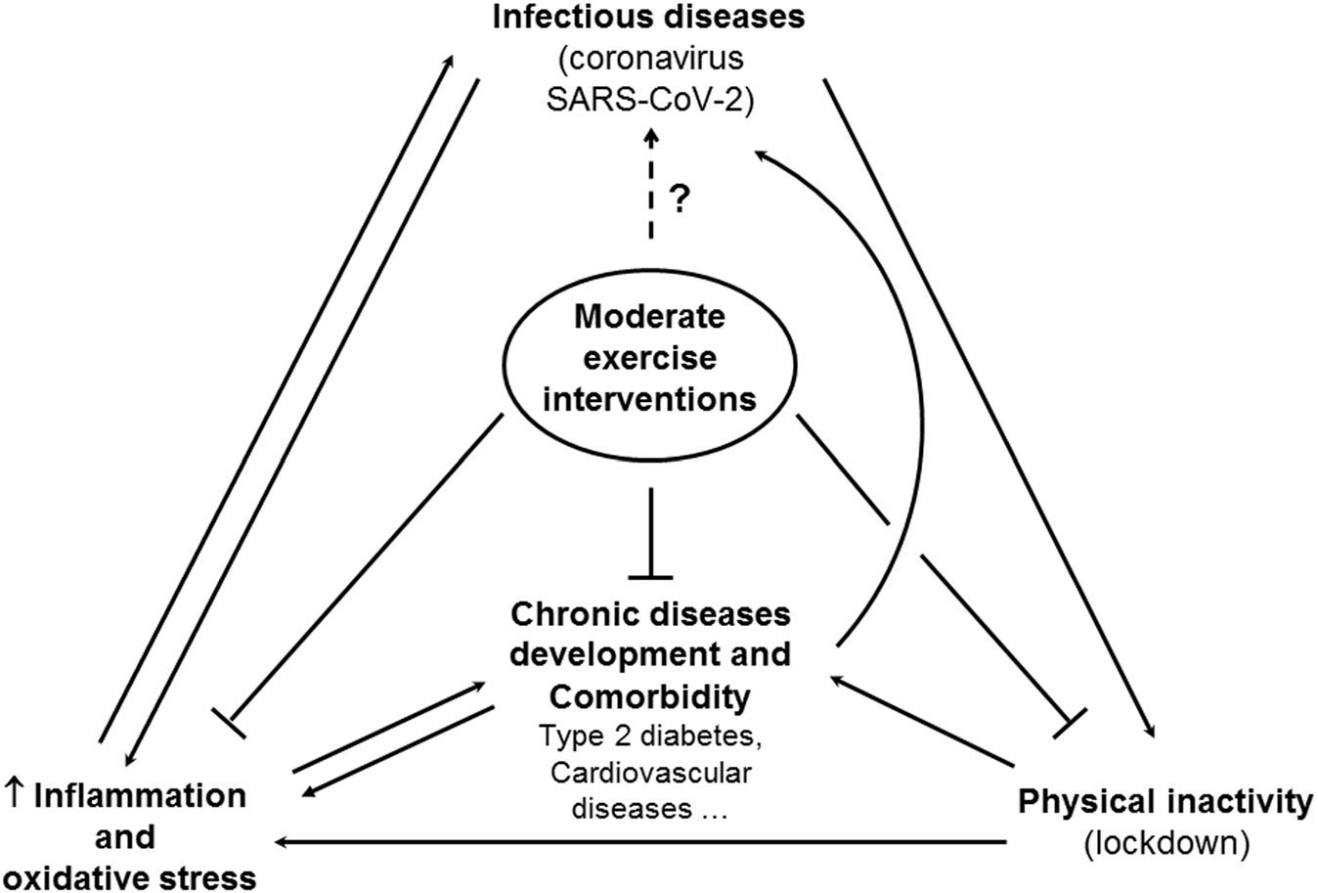
Schematic diagram representing known and hypothetical links between moderate exercise interventions and infectious diseases including COVID-19, inflammation, oxidative stress, and chronic diseases. Inflammation and oxidative stress are increased by viral infection, type 2 diabetes mellitus, cardiovascular diseases, and physical inactivity enacted by lockdown. Increased inflammation and oxidative stress may contribute to the susceptibility of both chronic disease like type 2 diabetes mellitus and infectious diseases. Inflammation may be the link between chronic diseases development and comorbidity and increased susceptibility to coronavirus. Moderate exercise interventions stimulate anti-inflammatory and antioxidant response, and prevent many chronic diseases development and comorbidity.
